# Association Between Patient-Provider Relationship Quality and Colorectal Cancer Screening Uptake Among US Adults

**DOI:** 10.7759/cureus.111352

**Published:** 2026-06-23

**Authors:** Emmanuel Abrefah, Matthew Asare, Elizabeth Kwon, Theophilus A Bediako, Kemea Perry

**Affiliations:** 1 Public Health, Baylor University, Waco, USA; 2 Statistics, Baylor University, Waco, USA

**Keywords:** colorectal cancer, healthcare communication, health disparities, patient-provider relationship, preventive care, screening uptake, shared decision-making

## Abstract

Patient-provider relationship (PPR) quality may influence colorectal cancer (CRC) screening uptake, yet national-level evidence remains limited. This study examined the association between PPR quality and CRC screening among US adults. This retrospective cross-sectional study used nationally representative data from the Health Information National Trends Survey (HINTS) 2018-2020 cycles. The analytic sample included 7,732 adults aged 45-75 years. Weighted descriptive analyses, chi-square tests, and multivariable logistic regression were conducted. Overall, 5,771 (76.2%) participants reported ever having completed a CRC screening test. Screening prevalence was 75.5% (n = 3,999) during the pre-COVID period and 77.9% (n = 1,772) during the COVID period. Screening uptake was lowest among adults aged 45-54 years (n = 908, 47.7%), compared with adults aged 55-64 years (n = 2,252, 81.7%) and 65-75 years (n = 2,611, 89.6%). Most respondents reported high PPR quality (n = 5,248, 82.8%). After adjustment, lower PPR quality was associated with reduced CRC screening uptake (adjusted odds ratio (aOR) = 0.72, 95% confidence interval (CI): 0.59-0.86). Lower emotional support (aOR = 0.72, 95% CI: 0.59-0.89) and reduced shared decision-making (aOR = 0.71, 95% CI: 0.56-0.89) were also associated with decreased screening uptake. Asian (aOR = 0.39, 95% CI: 0.17-0.87) and Hispanic (aOR = 0.34, 95% CI: 0.22-0.53) respondents reported lower PPR quality than non-Hispanic Whites. Lower PPR quality was associated with decreased CRC screening uptake. Strengthening supportive and culturally responsive patient-provider interactions may improve CRC screening participation and reduce disparities in preventive cancer care.

## Introduction

Colorectal cancer (CRC) is a major global public health challenge caused by the uncontrolled growth of abnormal cells in the colon or rectum. In the United States, CRC remains one of the leading causes of cancer-related morbidity and mortality, ranking third among men, fourth among women, and second overall when both sexes are combined [1]. In 2025, approximately 154,270 new cases and 52,900 deaths are projected nationwide [2]. Globally, CRC accounted for more than 2.17 million new cases and 1.09 million deaths in 2019, with incidence rates rising among younger adults, particularly in high-income countries [3]. In the United States alone, direct medical costs exceed $24 billion annually, representing nearly 12% of total cancer treatment expenditures [4]. These figures highlight the urgent need for effective prevention and early detection strategies, especially in increasing screening participation.

Despite its high burden, CRC is largely preventable. More than half of CRC cases and deaths are linked to modifiable lifestyle factors, including smoking, unhealthy diet, alcohol use, physical inactivity, and obesity [5,6]. Regular screening and timely intervention significantly reduce both incidence and mortality. For example, undergoing a colonoscopy every 10 years can lower CRC mortality by up to 73% [7]. The US Preventive Services Task Force (USPSTF) recommends several screening options, including colonoscopy, fecal immunochemical tests (FIT), stool DNA testing, and CT colonography. Early detection is critical, as five-year survival rates decline sharply from 91% for localized diseases to 13% for distant-stage diagnoses [2]. Although guidelines advise beginning screening at age 45 [2], screening uptake remains suboptimal. In 2023, only 63.5% of adults aged 45-75 were up to date with recommended screening [8], falling 10.3% points below the Healthy People 2030 targeted goal of 72.8% [9]. Significant disparities in CRC screening persist across racial, ethnic, and socioeconomic groups. For example, screening rates remain low among Asian Americans (approximately 50%), uninsured individuals (21%), and adults aged 45-49 (20%) [10]. Individuals with lower educational attainment also show disproportionately lower screening rates [10]. Multiple factors contribute to these disparities, including limited awareness of screening guidelines [11], lack of provider recommendation [12-14], and access-related challenges such as cost, transportation, and availability of services [14,15]. The COVID‑19 pandemic significantly disrupted colorectal cancer (CRC) screening, with a study reporting declines of 28%-100% globally during the early pandemic due to reduced healthcare capacity and postponed non‑urgent procedures [16]. Leveraging the patient-provider relationship (PPR) approach presents an opportunity to enhance CRC screening uptake by positively influencing screening behaviors.

Theoretical framework

The patient-provider relationship (PPR) is a key determinant of health behavior change and preventive care use [17-20]. High-quality PPR includes effective communication, trust, shared decision-making, and cultural sensitivity. Clear, empathetic, and two-way communication improves patients’ understanding of health risks and the benefits of colorectal cancer (CRC) screening [17]. Trust encourages openness, engagement, and adherence to medical advice [21]. Shared decision-making empowers patients by involving them in setting goals and selecting appropriate interventions, increasing accountability for their health [18]. Understanding how patient-provider relationship quality interacts with sociodemographic factors to influence colorectal cancer screening is essential for advancing equity in cancer prevention. However, national-level research on these associations remains limited [22]. This study examined the relationship between PPR quality and CRC screening uptake in a nationally representative US sample. Specifically, the objectives were to (1) examine the association between PPR quality and CRC screening uptake among US adults aged 45-75 years; (2) assess racial and ethnic disparities in PPR quality and CRC screening uptake; and (3) evaluate differences in CRC screening uptake and PPR quality between the pre-COVID (2018-2019) and COVID-period (2020) survey cycles.

## Materials and methods

Study design

We conducted a retrospective, population-level, analytical cross-sectional study using data from the Health Information National Trends Survey (HINTS) [23], administered by the National Cancer Institute (NCI). HINTS is a nationally representative survey designed to collect information on health communication, information-seeking behaviors, and cancer-related knowledge and perceptions among US adults. We utilized the 2018, 2019, and 2020 datasets, as they represent the most recent pre-pandemic and early-pandemic cycles that include measures of colorectal cancer (CRC) screening, patient-provider communication, and relevant covariates. The pooled datasets were used to examine associations between PPR quality and CRC screening uptake, as well as to compare outcomes between the pre-COVID period (2018-2019) and the early COVID period (2020).

Study population

HINTS surveys non-institutionalized US adults aged 18 years and older. For this study, the analytic sample was restricted to adults aged 45-75 years. Although the HINTS survey cycles analyzed (2018-2020) preceded the 2021 US Preventive Services Task Force (USPSTF) recommendation lowering the starting age for average-risk colorectal cancer (CRC) screening from 50 to 45 years, adults aged 45-49 years were included to align the analysis with current CRC screening recommendations and contemporary prevention priorities. Inclusion of this age group also allowed examination of screening behaviors among younger adults who may have undergone screening because of elevated risk, family history, physician recommendation, or evolving clinical practice patterns.

Across the three survey cycles, 2018 (n = 3,504), 2019 (n = 5,438), and 2020 (n = 3,865), the combined sample included 12,807 respondents. Participants were eligible if they provided complete data on the primary outcome (ever had CRC screening) and key predictor variables (patient-provider relationship (PPR) measures). Respondents with missing outcome or predictor data were excluded.

Measures

Independent Variables

**Figure 1:**
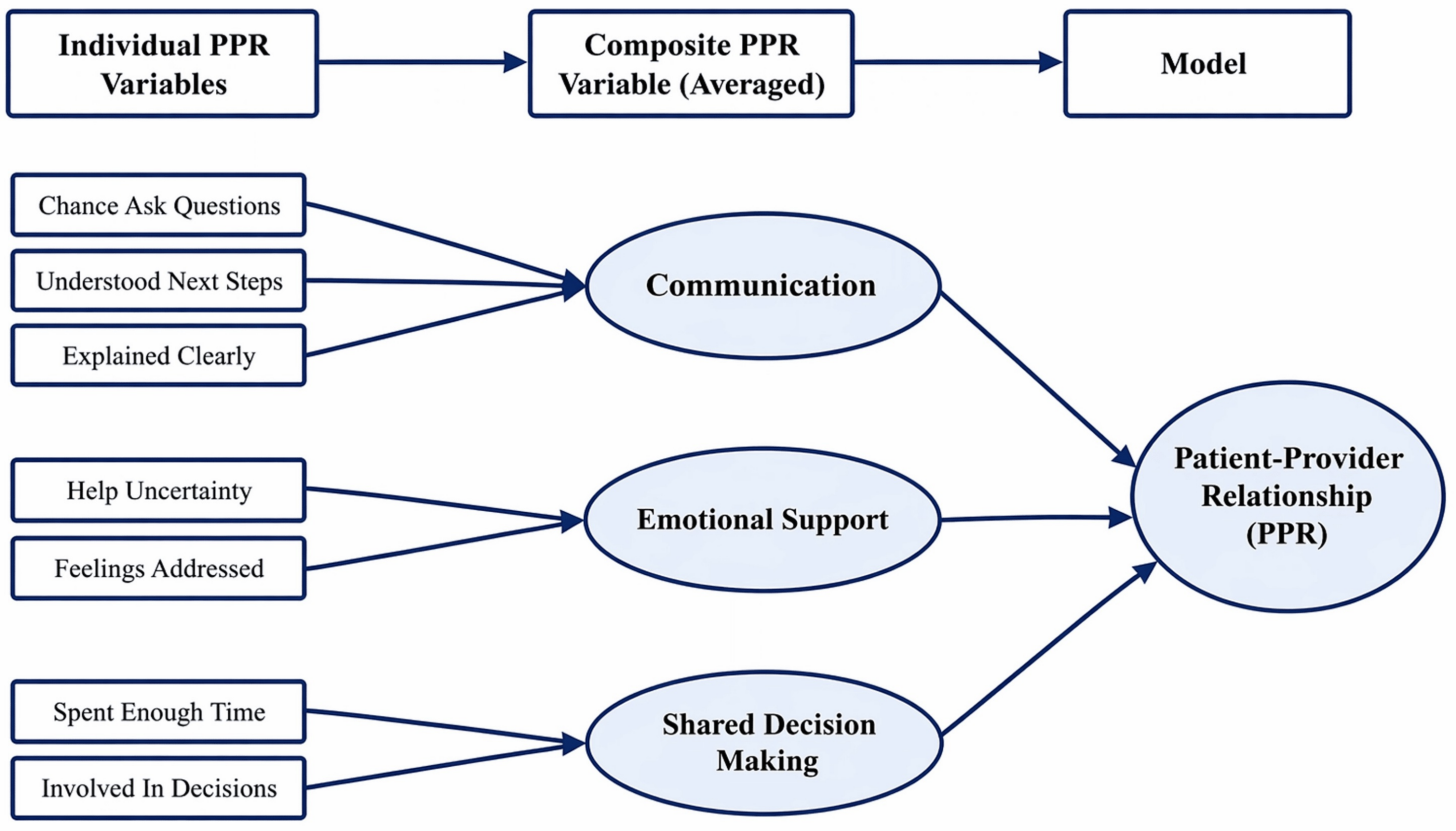
Path diagram displaying the computation of composite PPR variables This figure was created by the authors using Microsoft PowerPoint (Microsoft Corp., Redmond, WA). PPR: patient-provider relationship

The independent variables were derived from PPR subscales reflecting communication, emotional support, and shared decision-making (Figure 1). The communication subscale (three items) assessed clarity of explanations, opportunities to ask questions, and confirmation of understanding (Cronbach’s α = 0.87). The emotional support subscale (two items) measured acknowledgment of feelings and assistance in coping with uncertainty (α = 0.83). The shared decision-making subscale (two items) evaluated time spent and involvement in care decisions (α = 0.80). For each PPR domain, responses to the component items were combined to create a composite measure, with higher scores indicating higher perceived quality of patient-provider interactions. Items were rated on a 4-point Likert scale and dichotomized into high quality (“always/usually”) versus low quality (“sometimes/never”).

Dependent Variables

The primary dependent variable was self-reported history of colorectal cancer (CRC) screening. Respondents were classified as having undergone CRC screening if they reported ever completing a colonoscopy, sigmoidoscopy, or stool blood test to check for colon cancer. The outcome was coded as a binary variable (yes/no). To examine disparities, PPR subscales were also analyzed as outcomes with race/ethnicity as a predictor.

Covariates

Key demographic variables were included as covariates: age group, gender, annual household income, race/ethnicity, marital status, and education level. Each variable was categorized to support meaningful interpretation in the multivariate analyses.

Statistical analysis

Following the HINTS recommended analytic guidelines, we applied the appropriate sampling weights, cluster, and strata variables to generate weighted, population-based estimates for the included demographic characteristics, PPR variables, and CRC screening uptake. This approach ensured the national representativeness of the findings. Descriptive and bivariate analyses summarized sample characteristics and examined associations between PPR and CRC screening. Multivariate logistic regression was utilized to estimate both adjusted (aOR) and unadjusted odds (OR) of screening while controlling for potential confounders. To assess associations between colorectal cancer screening status (yes/no) and demographic variables, chi-square tests of independence were performed. Additionally, chi-square test of independence were conducted using combined pre-COVID-19 pandemic (2018-2019) and during pandemic (2020) data to assess COVID-19 impact. A complete case analysis was performed. For each statistical test conducted, observations with missing values for the relevant variable(s) were excluded from that specific analysis. No imputation procedures were performed. All analyses were performed using SAS 9.4 (SAS Inc., Cary, NC), with significance set at p < 0.05.

## Results

Demographic characteristics

The final analytic sample included 7,732 respondents, representing a weighted population of approximately 128.7 million US adults aged 45-75 years. Most participants were aged 55-64 years (n = 2,802, 36.24%) or 65-75 years (n = 2,979, 38.53%). Slightly more than half were female (n = 4,232, 56.46%), and 4,186 (54.88%) were married. The majority had at least some college education (n = 5,637, 72.90%), and over one-third (n = 2,602, 33.65%) reported annual household incomes of $75,000 or higher. Most respondents had health insurance coverage (93.91% weighted). Racial and ethnic distribution was predominantly non-Hispanic White (n = 4,472, 57.84%), followed by non-Hispanic Black (n = 1,078, 13.94%), Hispanic (n = 1,002, 12.96%), non-Hispanic Asian (n = 266, 3.44%), and non-Hispanic Other (n = 236, 3.05%). Overall, 5,771 (74.64%) reported ever completing a colorectal cancer (CRC) screening test (Table 1).

**Table 1:** Unweighted and weighted frequencies of sociodemographic characteristics and colorectal cancer screening uptake among US adults For descriptive analyses of each variable, missing data were handled using case-wise deletion. Unweighted percentages (%): calculated as the number of respondents in each category divided by the total unweighted sample size (7,732). Weighted percentages (%): these are population-based estimates generated by applying the HINTS sampling weights, cluster, and strata variables. They represent the estimated proportion of the US adult population (approximately 128.7 million) in each category, accounting for the complex survey design. HINTS: Health Information National Trends Survey, BMI: body mass index

Parameters	Unweighted (n = 7,732, 100%)		Weighted (n = 128,665,867, 100%)
	Number	%	%
Age category			
45-54	1,951	25.23	44.70
55-64	2,802	36.24	31.46
65-75	2,979	38.53	23.84
Year category			
2018-2019	5,400	69.84	80.83
2020	2,332	30.16	19.17
Sex			
Male	3,263	42.2	49.49
Female	4,232	54.73	50.51
Missing	237	3.07	
Married			
Yes	4,186	54.14	65.31
No	3,442	44.52	34.69
Missing	104	1.34	
Education			
Less than high school	544	7.04	7.67
High school graduate	1,456	18.83	24.69
Some colleges	2,383	30.82	40.68
College graduate	3,254	42.08	26.96
Missing	95	1.23	
Annual income			
Less than $20,000	1,323	17.11	17.48
$20,000 to less than $35,000	890	11.51	10.58
$35,000 to less than $50,000	917	11.86	12.91
$50,000 to less than $75,000	1,235	15.97	17.23
≥$75,000	2,602	33.65	41.80
Missing	765	9.89	
Health insurance			
Yes	7,295	94.35	93.91
No	331	4.28	6.09
Missing	106	1.37	
BMI			
Underweight	99	1.28	1.00
Normal weight	2,327	30.09	30.45
Overweight	2,191	28.34	29.29
Obese	2,925	37.83	39.26
Missing	190	2.46	
Race/ethnicity			
Non-Hispanic White	4,472	57.84	67.38
Non-Hispanic Black	1,078	13.94	11.97
Hispanic	1,002	12.96	14.11
Non-Hispanic Asian	266	3.44	4.22
Non-Hispanic Other	236	3.05	2.32
Missing	678	8.77	
Ever had cancer			
Yes	1,361	17.6	11.45
No	6,348	82.1	88.55
Missing	23	0.3	
Colon cancer diagnosis			
Yes	63	0.81	3.39
No	1,282	16.58	96.61
Missing	6,387	82.6	
Ever tested for colon cancer			
Yes	5,771	74.64	66.84
No	1,802	23.3	33.16
Missing	159	2.06	

Main results

Predictors of Colorectal Cancer Screening Uptake

Respondents reporting ever having undergone CRC screening were 5,771 (76.2%); however, when stratified by time period, it was 3,999 (75.5%) in the pre-COVID period and 1,772 (77.9%) during the COVID period. CRC screening rates significantly varied across different age groups, with the lowest rates reported among adults aged 45-54 (n = 908, 47.7%), followed by those aged 55-64 (2,252, 81.7%) and 65-75 (2,611, 89.6%). Screening rates were higher among married versus unmarried individuals (n = 3,193, 78.0% versus n = 2,521, 74.8%), those with a college degree versus less than a high school education (n = 2,537, 79.1% versus n = 345, 65.3%), and high-income earners (≥$75,000) compared with lower-income earners (n = 1,999, 77.8% versus n = 928, 72.2%) (Table 2).

**Table 2:** Bivariate analysis summarizing sample characteristics and associations with CRC screening Chi-square test For each chi-square test, cases with missing data on the variables involved were excluded. The proportions (percentages) in this table are computed as row percentages; that is, for each category of a given characteristic, the percentage represents the proportion of people in that category who said “No” or “Yes” to colorectal cancer screening. CRC: colorectal cancer, BMI: body mass index

Parameters	Colorectal cancer screening (number (%))		Chi-square
	Yes	No	
Overall	5,771 (76.2)	1,802 (23.8)	
Year category			X^2^ (1) = 5.09, p = 0.025
Pre-COVID (2018-2019)	3,999 (75.5)	1,299 (24.5)	
During COVID (2020)	1,772 (77.9)	503 (22.1)	
Age			X^2^ (2) = 1183.24, p < 0.001
45-54	908 (47.7)	994 (52.3)	
55-64	2,252 (81.7)	504 (18.3)	
65-75	2,611 (89.6)	304 (10.4)	
Gender			X^2^ (1) = 1.42, p = 0.233
Male	2,485 (76.9)	747 (23.1)	
Female	3,149 (75.7)	1,011 (24.3)	
Marital status			X^2^ (1) = 14.78, p ≤ 0.001
Married	3,193 (77.5)	926 (22.5)	
Not married	2,521 (74.8)	848 (25.2)	
Educational level			X^2^ (3) = 59.18, p ≤ 0.001
Less than high school	345 (65.3)	183 (34.7)	
High school graduate	1,035 (72.8)	386 (27.2)	
Some colleges	1,803 (76.9)	541 (23.1)	
College graduate	2,537 (79.1)	669 (20.9)	
Annual income			X^2^ (4) = 26.22, p ≤ 0.001
Less than $20,000	928 (72.2)	357 (27.8)	
$20,000 to less than $35,000	645 (74.1)	225 (25.9)	
$35,000 to less than $50,000	677 (74.6)	231 (25.4)	
$50,000 to less than $75,000	966 (79.7)	246 (20.3)	
≥$75,000	1,999 (77.8)	572 (22.2)	
Health insurance			X^2^ (1) = 278.66, p ≤ 0.001
Yes	5,582 (78.0)	1,572 (22.0)	
No	121 (37.6)	201 (62.4)	
BMI			X^2^ (3) = 8.98, p = 0.030
Underweight	70 (72.9)	26 (27.1)	
Normal weight	1,697 (74.3)	587 (25.7)	
Overweight	1,669 (77.5)	485 (22.5)	
Obese	2,216 (77.3)	650 (22.7)	
Race/ethnicity			X^2^ (4) = 70.41, p ≤ 0.001
Non-Hispanic White	3,456 (78.3)	957 (21.7)	
Non-Hispanic Black	816 (78.4)	225 (21.6)	
Hispanic	673 (68.1)	315 (31.9)	
Non-Hispanic Asian	177 (67.3)	86 (32.7)	
Non-Hispanic Other	157 (67.4)	86 (32.6)	
Ever had cancer			X^2^ (1) = 125.08, p ≤ 0.001
Yes	1,880 (88.0)	161 (12.0)	
No	4,588 (73.7)	1,641 (26.3)	
Colon cancer diagnosis			X^2^ (1) = 3.01, p = 0.083
Yes	59 (95.2)	3 (4.8)	
No	1,111 (87.9)	153 (12.1)	

PPR as a Predictor of Colorectal Cancer Screening

**Figure 2:**
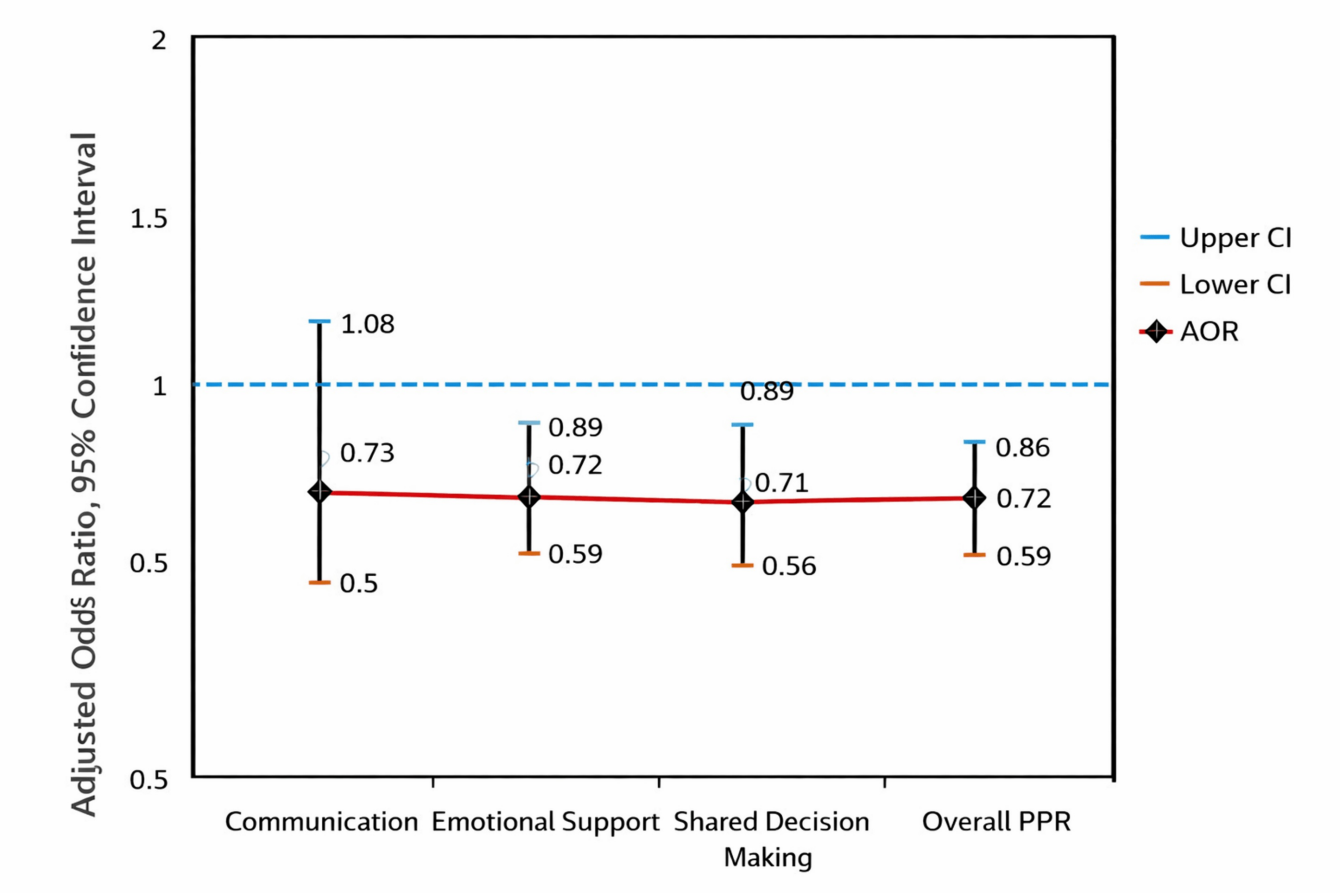
Association between PPR and colorectal cancer screening behavior using multivariable logistic regression analysis High-frequency PPR served as the reference category. High frequency indicates that patients consistently received communication, emotional support, and shared decision-making from healthcare providers. The model was adjusted for age, gender, marital status, education, and income. Emotional support, shared decision-making, and overall PPR were significantly associated with colorectal cancer screening behavior (p < 0.01), whereas communication was not statistically significant (p > 0.05). PPR: patient-provider relationship, AOR: adjusted odds ratio, CI: confidence interval

About 5,248 (82.8%) respondents reported high patient-provider relationship (PPR) quality; however, when stratified by time period, it was 3,562 (83.2%) in the pre-COVID period and 1,686 (81.9%) during the COVID period. After adjusting for covariates, lower overall PPR quality was significantly associated with reduced CRC screening (aOR = 0.72, 95% confidence interval (CI): 0.59-0.86). Specifically, lower emotional support (aOR = 0.72, 95% CI: 0.59-0.89) and shared decision-making (aOR = 0.71, 95% CI: 0.56-0.89) were significantly associated with decreased screening uptake, while communication alone was not statistically significant (Figure 2).

**Figure 3:**
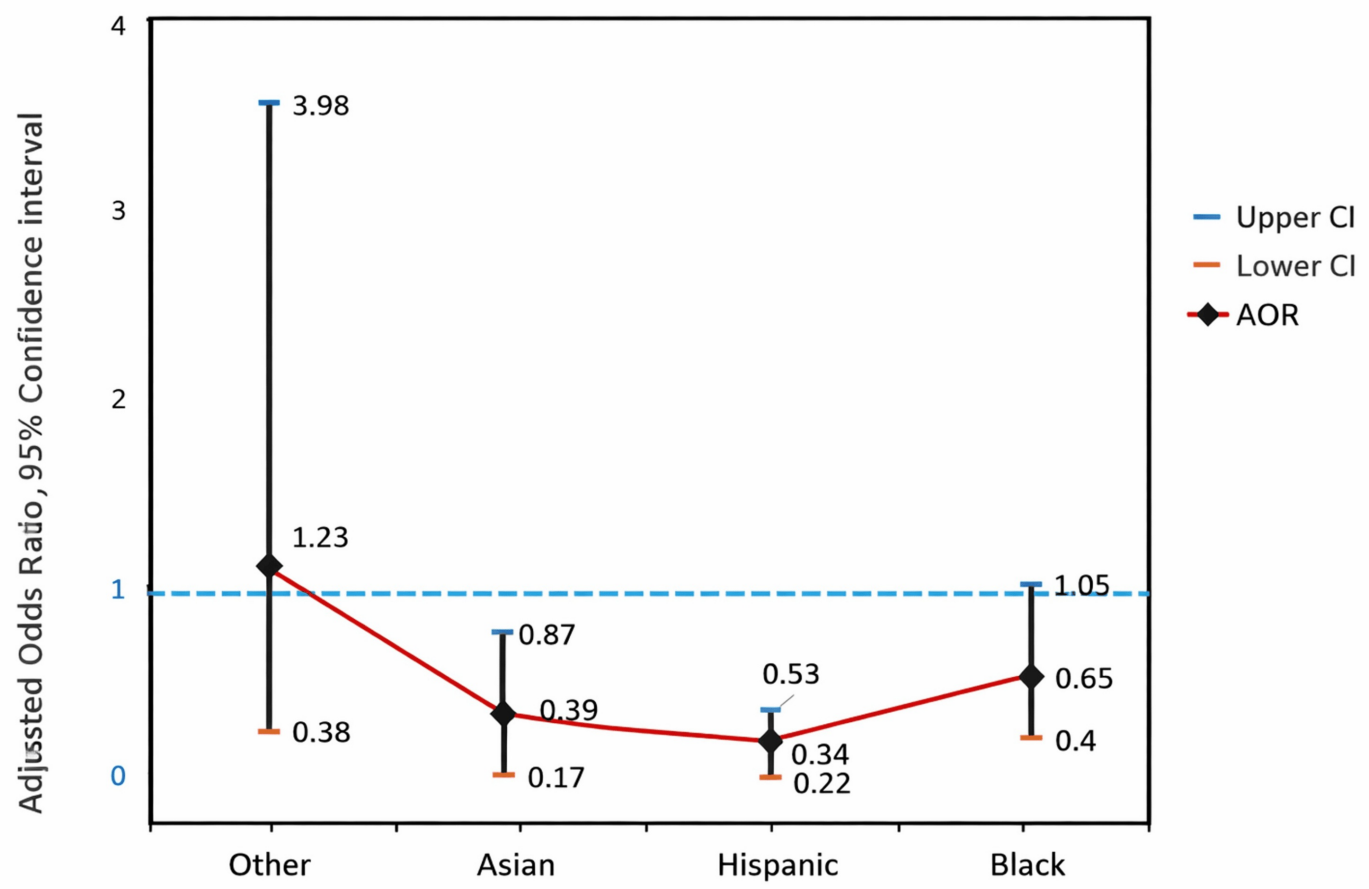
Association between race/ethnicity and overall PPR quality using multivariable logistic regression analysis Non-Hispanic White participants served as the reference category. The model was adjusted for age, sex, marital status, education, insurance status, BMI, and income. Asian participants (p < 0.01) and Hispanic participants (p < 0.001) had significantly lower odds of reporting high-quality PPR compared with non-Hispanic White participants. Associations for Black participants and those categorized as “Other” race/ethnicity were not statistically significant (p > 0.05). PPR: patient-provider relationship, AOR: adjusted odds ratio, CI: confidence interval

To examine racial and ethnic disparities in patient-provider relationship (PPR) quality, multivariable logistic regression models were conducted with overall PPR quality as the outcome and race/ethnicity as the primary predictor. After adjustment for age, sex, marital status, education, insurance status, BMI, and income, Asian respondents (aOR = 0.39, 95% CI: 0.17-0.87) and Hispanic respondents (aOR = 0.34, 95% CI: 0.22-0.53) had significantly lower odds of reporting high-quality PPR compared with non-Hispanic White respondents, who served as the reference group. No statistically significant differences were observed among Black respondents (aOR = 0.65, 95% CI: 0.40-1.05) or respondents categorized as other race/ethnicity (aOR = 1.23, 95% CI: 0.38-3.98). Figure 3 presents the adjusted odds ratios and corresponding 95% confidence intervals for these associations.

## Discussion

This study examined the association between patient-provider relationship (PPR) quality and colorectal cancer (CRC) screening uptake among US adult populations. The major findings indicate that the prevalence of self-reported history of CRC screening was high across survey cycles and that lower PPR quality was significantly associated with decreased CRC screening uptake.

In this nationally representative sample, the overall colorectal cancer (CRC) screening rate was 5,771 (76.2%), with a modest difference observed between respondents surveyed during the COVID period and those surveyed during the pre-COVID period (n = 1,772, 77.9% versus n = 3,999, 75.5%). Although many studies have documented substantial disruptions in CRC screening during the COVID-19 pandemic [16,24,25], the outcome used in this study reflects a self-reported history of ever having undergone CRC screening rather than screening within a defined time interval. Therefore, the observed differences between survey periods should not be interpreted as direct evidence of pandemic-related changes in screening behavior. Nonetheless, the findings suggest that the prevalence of prior CRC screening was similar across the survey cycles examined. Further research using measures of guideline-concordant or recent CRC screening is needed to better understand the impact of the COVID-19 pandemic on CRC screening utilization.

Consistent with established literature, screening rates varied substantially by age, with the lowest uptake among adults aged 45-54 years (n = 908, 47.7%), despite recent guideline updates recommending initiation of CRC screening at age 45 [26]. Notably, the study revealed significant racial and ethnic disparities in CRC screening. Non-Hispanic Asian individuals were 37% less likely to be screened compared to Non-Hispanic White individuals (aOR = 0.63, 95% CI: 0.46-0.86), while Non-Hispanic Black/African American individuals were 53% more likely to report screening (aOR = 1.53, 95% CI: 1.25-1.86). These differences may reflect a complex interplay of cultural beliefs, healthcare access, and experiences with the healthcare system. Although higher screening rates among Black/African American individuals are encouraging, efforts are still needed to ensure equitable access and outcomes across all racial and ethnic groups [27].

Importantly, PPR quality emerged as a predictor of CRC screening, even after adjusting for covariate variables. Although more than 5,248 (80%) respondents reported high PPR quality, lower overall PPR was independently associated with reduced screening uptake (aOR = 0.72, 95% CI: 0.59-0.86). Manne et al. [28] found that CRC screening intentions were significantly higher among patients who perceived their providers as attentive, informative, and collaborative. Similarly, Politi et al. [29] showed that PPR quality was positively associated not only with screening completion but also with patient satisfaction and trust in medical advice. The results of this study support and extend these findings, underscoring that quality interactions, characterized by clear communication, shared decision-making, and emotional responsiveness, may act as a behavioral catalyst for CRC screening. Although overall PPR quality was high, the communication domain demonstrated lower ratings than the emotional support and shared decision-making domains. The relatively lower ratings observed for communication may reflect factors not directly measured in HINTS, such as time constraints during clinical encounters, health literacy challenges, language barriers, or differences in patient expectations regarding communication. Future studies should explore these factors in greater detail to better understand barriers to effective patient-provider communication and their influence on preventive health behaviors.

Disparities in the quality of PPR were also evident in the study findings. Specifically, Asian (adjusted odds ratio (aOR) = 0.39, 95% CI: 0.17-0.87) and Hispanic participants (aOR = 0.34, 95% CI: 0.22-0.53) had substantially lower odds of reporting frequent, high-quality PPR compared to their non-Hispanic White counterparts. These disparities are particularly concerning, given the well-documented influence of PPR on engagement in preventive health behaviors, including CRC screening [30,31]. These findings raise critical questions about the cultural competence and inclusivity of provider communication in the US healthcare system. Previous studies have shown that minority patients are more likely to experience less participatory communication styles, reduced empathy, and diminished trust in healthcare providers, factors that collectively undermine the quality of the patient-provider relationship [32-34]. For example, Johnson et al. [35] found that African American and Hispanic patients were less likely to report feeling listened to or respected by their physicians, which in turn contributed to lower satisfaction with care and reduced screening uptake. Similarly, research by Saha et al. [36] highlighted how perceived discrimination and lack of cultural sensitivity in clinical interactions erode trust and discourage preventive care utilization among minority populations. These disparities in PPR quality may serve as an important mediating factor in observed disparities in CRC screening and other preventive health services. Enhancing the cultural responsiveness of healthcare communication, through provider training, patient-centered communication models, and improved access to language-concordant care, may be essential to improving both PPR quality and health equity [37-39].

Strengths and limitations

Several limitations of this study should be acknowledged. First, reliance on self-reported CRC screening data may introduce recall bias, as participants may have difficulty accurately remembering past screening behaviors. Additionally, the outcome measured a history of ever having undergone CRC screening and did not assess whether respondents were up to date with guideline-recommended screening intervals. Therefore, the findings should be interpreted as reflecting prior CRC screening experience rather than adherence to current screening recommendations. Social desirability bias could lead some individuals to overreport adherence to screening guidelines, especially if they perceive CRC screening as a socially or medically recommended behavior. Future studies utilizing electronic health records or claims data may provide more objective measures of CRC screening uptake. The cross-sectional design of this study limits the ability to establish causal relationships between PPR quality and CRC screening uptake. While strong associations were observed, it remains unclear whether better patient-provider interactions directly lead to increased screening or if individuals who are already inclined to participate in preventive healthcare are more likely to report positive PPR experiences. Longitudinal studies tracking patient-provider interactions over time could help clarify the directionality of this relationship. Moreover, while the study adjusted for major sociodemographic confounders, unmeasured factors such as healthcare access, provider characteristics, language barriers, and psychosocial influences may have impacted the results. In particular, language proficiency and language concordance between patients and providers were not assessed and may have influenced perceptions of communication quality among racially and ethnically diverse populations.

Nonetheless, this study has several notable strengths. One of its key strengths is the use of a large, nationally representative dataset, which ensures that the findings can be generalized to the broader US adult population. The survey’s rigorous sampling methodology enhances the external validity of the study, making it a valuable resource for examining trends in CRC screening behaviors at a national level. Additionally, this study takes a comprehensive approach by examining multiple dimensions of PPR communication, emotional support, and shared decision-making. Moreover, by utilizing survey-weighted statistical methods, this study effectively addresses the complex sampling design of HINTS, thereby minimizing potential biases arising from survey non-response patterns. These methodological strengths significantly enhance the study’s contribution to the literature on CRC screening and highlight the critical importance of promoting effective patient-provider interactions in preventive healthcare.

Public health implications

These findings have important public health implications. Enhancing PPR during healthcare encounters may be an effective strategy to improve CRC screening rates, thereby reducing the burden of CRC morbidity and mortality. Interventions aimed at training healthcare providers in communication skills, cultural competency, and patient-centered care could substantially impact screening uptake. Additionally, health systems could integrate communication quality assessments into routine practice to identify and address gaps in patient engagement. Given that disparities in CRC screening persist by race/ethnicity, targeted efforts to strengthen PPR in underserved populations are particularly critical. Addressing relational barriers could complement structural interventions (e.g., increasing screening access) to achieve more equitable cancer prevention outcomes.

## Conclusions

Colorectal cancer screening rates were high among respondents in this nationally representative sample; however, important socioeconomic, racial/ethnic, and age-related disparities persisted. Lower PPR quality, particularly reduced emotional support and shared decision-making, was significantly associated with decreased screening uptake. Racial and ethnic differences in perceived relationship quality further highlight ongoing inequities. Strengthening supportive, culturally responsive patient-provider interactions may be essential to improving CRC screening participation and advancing health equity.
